# Multiple COVID-19 Outbreaks Linked to a Wedding Reception in Rural Maine — August 7–September 14, 2020

**DOI:** 10.15585/mmwr.mm6945a5

**Published:** 2020-11-13

**Authors:** Parag Mahale, Craig Rothfuss, Sarah Bly, Megan Kelley, Siiri Bennett, Sara L. Huston, Sara Robinson

**Affiliations:** ^1^Maine Center for Disease Control and Prevention, Augusta, Maine; ^2^Epidemic Intelligence Service, CDC; ^3^University of Southern Maine, Portland, Maine.

Large indoor gatherings pose a high risk for transmission of SARS-CoV-2, the virus that causes coronavirus disease 2019 (COVID-19), and have the potential to be super-spreading events (*1*,*2*). Such events are associated with explosive growth, followed by sustained transmission (*3*). During August 7–September 14, 2020, the Maine Center for Disease Control and Prevention (MeCDC) investigated a COVID-19 outbreak linked to a wedding reception attended by 55 persons in a rural Maine town. In addition to the community outbreak, secondary and tertiary transmission led to outbreaks at a long-term care facility 100 miles away and at a correctional facility approximately 200 miles away. Overall, 177 COVID-19 cases were epidemiologically linked to the event, including seven hospitalizations and seven deaths (four in hospitalized persons). Investigation revealed noncompliance with CDC’s recommended mitigation measures. To reduce transmission, persons should avoid large gatherings, practice physical distancing, wear masks, stay home when ill, and self-quarantine after exposure to a person with confirmed SARS-CoV-2 infection. Persons can work with local health officials to increase COVID-19 awareness and determine the best policies for organizing social events to prevent outbreaks in their communities.

## Investigation and Results

On August 12, 2020, MeCDC received laboratory reports of two persons who received positive SARS-CoV-2 polymerase chain reaction (PCR) test results from nasopharyngeal swab specimens. Both persons reported attending a wedding reception on August 7, and both experienced onset of fever, cough, and sore throat on August 11. Three more persons who reported attending the same reception received positive SARS-CoV-2 test results the next day, prompting initiation of an outbreak investigation by MeCDC on August 14.

MeCDC used the Council of State and Territorial Epidemiologists’ COVID-19 case definitions (*4*). Confirmed cases were defined by receipt of a positive SARS-CoV-2 PCR test result, and probable cases were defined by the reported presence of COVID-19–compatible symptoms and epidemiologic linkage to a confirmed case, without laboratory testing (*4*). Close contact was defined as being within 6 feet of a person with COVID-19 for at least 15 minutes (*4*). MeCDC defines a COVID-19 outbreak as the occurrence of three or more confirmed cases within 14 days in persons from different households, with an epidemiologic link to a single facility or event. MeCDC investigators interviewed patients using a standardized questionnaire*; entered data on demographic characteristics, onset date, symptoms, and relevant exposures into the National Electronic Disease Surveillance System Base System; and enrolled close contacts in Sara Alert,^†^ an automated, web-based, symptom-monitoring tool (*5*).

Primary cases were defined as confirmed or probable cases in persons present at the reception. Through contact tracing, MeCDC identified secondary and tertiary cases (close contacts of primary and secondary cases, respectively). MeCDC developed transmission chains using MicrobeTrace (version 0.6.1; CDC)^§^ and performed descriptive statistics using SAS software (version 9.4; SAS Institute). This activity was reviewed by CDC and was conducted consistent with applicable federal law and CDC policy.^¶^

The reception, attended by 55 persons, was held on August 7 in a rural Maine town located in county A. The town had a total population of approximately 4,500 persons and no previously reported COVID-19 cases. COVID-19 incidence in county A before the reception was 97 cases per 100,000 persons. The bride, groom, and groom’s family (seven persons) traveled from California to Maine on August 6. In compliance with the governor of Maine’s executive order,** because they had received negative SARS-CoV-2 test results shortly after arrival, they were not required to quarantine for 14 days. The index patient was a Maine resident and a wedding reception guest who reported onset of fever, runny nose, cough, and fatigue on August 8 and received a positive SARS-CoV-2 test result on August 13. During August 8–14, 24 persons who received positive SARS-CoV-2 PCR test results in county A reported having attended the event, prompting a health inspection of the facility to investigate compliance with Maine’s COVID-19 guidelines.^††^

The reception was held at a lodging establishment in county A that had an attached restaurant and four dining areas, including the event room, breakfast room, bar, and an open deck. Guests were seated indoors in the event room, which had 10 tables, with 4–6 guests seated around each table. The total number of wedding guests (55) exceeded Maine’s 50-person limit for indoor gathering in a shared space. Facility staff members had conducted temperature checks for all guests at the facility entrance; these were reported as normal. Although the facility had signs posted at the entrance instructing visitors to wear masks, guests did not comply with this requirement nor maintain a physical distance of ≥6 feet, and staff members did not enforce these measures; all staff members wore masks. The facility did not collect contact information from guests. A member of the wedding party informed MeCDC of the total number of guests but did not provide their contact information. MeCDC investigators linked cases to the event by backward tracing,^§§^ in which persons with confirmed or probable COVID-19 were interviewed to determine whether they had attended an event or were a close contact of a person who had received a diagnosis of COVID-19 ≤14 days before their symptom onset or testing date.

## Wedding Guests, Facility Staff Members, and Their Community Contacts

By August 20, MeCDC identified 30 primary cases (Figure). Confirmed SARS-CoV-2 infection was identified in 27 (49.1%) of 55 wedding guests. The other three primary cases occurred in a staff member at the venue, a vendor, and a patron dining at the venue who was not a wedding guest. The facility manager informed MeCDC that among the 30 staff members, 23 (76.7%) received negative SARS-CoV-2 test results, five (16.7%) self-quarantined without testing, and two (6.7%) received a positive test result (one primary case, one secondary case). MeCDC subsequently identified an additional 17 secondary and 10 tertiary cases (Supplementary Figure, https://stacks.cdc.gov/view/cdc/96482). Fifty-one (89.5%) patients were symptomatic, and 51 (89.5%) cases were confirmed (Table). The median age was 51 years, seven (12.3%) patients were aged ≥75 years, and the majority of cases occurred in women (57.9%). Among four persons hospitalized, one died; all four hospitalized patients were aged ≥75 years, had underlying medical conditions, and were not wedding guests.

**FIGURE:**
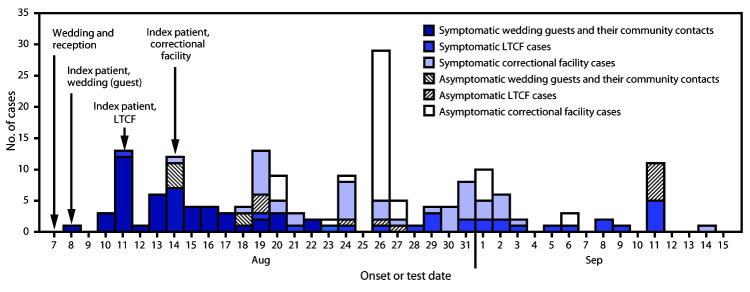
Distribution of COVID-19 cases (N = 177) linked to a rural wedding reception, by date of onset or test* — Maine, August 7–September 14, 2020 **Abbreviations: **COVID-19 = coronavirus disease 2019; LTCF = long-term care facility. * Date of test was used for asymptomatic cases.

**TABLE:** Demographic and clinical characteristics of COVID-19 cases associated with a wedding reception event and linked outbreaks (N = 177) — Maine, August 7–September 14, 2020

Characteristic	Outbreak cases/persons no./total no. (%)		
	Wedding reception attendees and their community contacts*	Long-term care facility	Correctional facility
**Total no. of cases**	57	38	82
**Case classification, no. (%) of all outbreak cases)**	Primary: 30 (52.6)	Staff member: 14 (36.8)	Staff member: 18 (22.0)
	Secondary: 17 (29.8)	Resident: 24 (63.2)	Incarcerated person: 48 (58.5)
	Tertiary: 10 (17.5)	—	Household contact of staff member:16 (19.5)
**Case status**			
Symptomatic confirmed	45/57 (79.0)	26/38 (68.4)	33/82 (40.2)
Symptomatic probable	6/57 (10.5)	0 (—)	9/82 (11.0)
Asymptomatic confirmed	6/57 (10.5)	12/38 (31.6)	40/82 (48.8)
**Age group, yrs, median (IQR)**	51 (27–63)	68 (46–83)	35 (29–48)
<18	11/57 (19.3)	1/38 (2.6)	7/82 (8.5)
18–29	5/57 (8.8)	3/38 (7.9)	17/82 (20.7)
30–59	22/57 (38.6)	8/38 (21.1)	52/82 (63.4)
60–75	12/57 (21.1)	10/38 (26.3)	6/82 (7.3)
≥75	7/57 (12.3)	16/38 (42.1)	0 (—)
**Sex**			
Male	24/57 (42.1)	6/38 (15.8)	63/82 (76.8)
Female	33/57 (57.9)	32/38 (84.2)	19/82 (23.2)
**Symptoms^†^**	51/57 (89.5)	26/38 (68.4)	42/82 (51.2)
No. of signs/symptoms per patient, median (IQR)	4 (2–6)	6 (4–9)	5 (3–7)
Duration of symptoms in days, median (IQR)	8 (6–10)	11 (1–12)	8 (6.5–10)
**Underlying medical conditions^†^**	19/57 (33.3)	26/38 (68.4)	43/82 (52.4)
**No. of cases hospitalized**	4/57 (7.0)	3/38 (7.9)	0 (—)
**No. of deaths**	1/57 (1.8)	6/38 (15.8)	0 (—)

**Abbreviations:** COVID-19 = coronavirus disease 2019; IQR = interquartile range.

* Includes the index cases at the long-term care and correctional facilities.

^†^ Each case could have multiple symptoms or underlying medical conditions.

One event attendee with COVID-19 (patient A1) reported cough onset on August 10 and attended an in-person school meeting the same day. Two school staff members subsequently received diagnoses of COVID-19 on August 14 and 17. Local schools delayed reopening by 2 weeks while all exposed staff members completed isolation or quarantine.

## Long-Term Care Facility Outbreak

After the reception, one of the guests (patient A2) had a close interaction with patient B1 (A2’s parent), a health care worker at long-term care facility (LTCF) A (Supplementary Figure, https://stacks.cdc.gov/view/cdc/96482) on August 8 and 9. Patient B1 reported fever, chills, cough, myalgia, runny nose, and headache on August 11, but nevertheless worked on August 11 and 12; patient B1 was tested for SARS-CoV-2 on August 13 and received a positive result on August 18.

MeCDC recommended universal testing for all LTCF A residents and staff members, which was conducted on August 19, after which five additional cases were detected in four residents and one staff member. An investigation at the facility was initiated on August 21. During August 19–September 11, MeCDC identified 38 additional persons with confirmed SARS-CoV-2 infection at LTCF A (Table); in 14 (18.4%) of 76 staff members and 24 (54.5%) of 44 residents. These persons accounted for 36.8% (staff members) and 63.2% (residents) of respective facility-associated cases. Compared with LTCF A staff member patients, more resident patients were aged ≥75 years (66.7% versus 0%) and had at least one underlying medical condition (87.5% versus 35.7%); however, symptoms were more prevalent in staff members (92.9%) than in residents (54.2%). Three residents were hospitalized, and six died; all decedents were aged ≥60 years and had underlying medical conditions.

## Correctional Facility Outbreak

One wedding guest (patient A3) was a correctional facility staff member and reported onset of cough, myalgia, runny nose, sore throat, and a new onset loss of taste sensation on August 14 (Supplementary Figure, https://stacks.cdc.gov/view/cdc/96482). During August 15–19, patient A3 worked daily 8-hour shifts in two separate correctional facility housing units while symptomatic. On August 19, four staff members, including patient A3, received confirmation of COVID-19 diagnoses, which led MeCDC to initiate an investigation at this facility.

By September 1, 18 additional staff members and 46 incarcerated persons had received positive SARS-CoV-2 test results (Figure). MeCDC and the Maine Department of Corrections visited the correctional facility on September 4 to assess mitigation measures. The facility had not implemented daily symptom screening for staff members or enforced regular use of masks after the first case was identified. During August 27–September 10, the facility implemented COVID-19 mitigation measures consistent with CDC guidelines for correctional facilities.^¶¶^

In addition to patient A3, MeCDC identified 82 confirmed COVID-19 cases at the correctional facility (Table), including cases in 18 (41.9%) of 43 staff members, 48 (41.4%) of 116 incarcerated persons, and 16 household contacts of staff members; these persons accounted for 22.0%, 58.5%, and 19.5% of facility-associated cases, respectively. Most patients were men (76.8%, including all incarcerated persons) and aged 30–59 years (76%). More staff members than incarcerated persons were symptomatic (83.3% versus 22.9%). No hospitalizations or deaths occurred.

## Discussion

A wedding reception in a small rural town was the likely source of COVID-19 outbreaks in the local community, an LTCF, and a correctional facility, leading to 177 cases, seven hospitalizations, and seven deaths, highlighting the importance of adhering to recommended mitigation measures even in communities where transmission rates are low. None of the persons who were hospitalized or died had attended the event. Robust case investigation and contact tracing allowed seemingly disparate outbreaks to be epidemiologically linked to the event. Index patients at the LTCF and the correctional facility both worked while symptomatic, underscoring the importance of staying home when ill.

Community gatherings such as weddings, birthday parties, church events, and funerals have the potential to be SARS-CoV-2 super-spreading events (*1*–*3*). Increased transmission risk at such events might result from failure to maintain physical distancing and inconsistent use of masks. Transmission risk is further increased when events are held indoors.*** Findings from this investigation also demonstrate that, in addition to asymptomatic and presymptomatic transmission (*6*,*7*), lack of adherence to CDC’s COVID-19 guidelines to stay home from work while symptomatic is an important contributor to spread of SARS-CoV-2 infection.^†††^

The findings in this report are subject to at least two limitations. First, a list of reception attendees was not available, and some infected persons might have been missed. Therefore, MeCDC likely undercounted cases of illness that were linked to the event, and the attack rate for the reception guests is thus a conservative estimate. Second, staff members at the LTCF and correctional facility possibly had exposures outside the facilities, and a definitive linkage of outbreaks at these facilities to the event was not possible in the absence of whole-genome sequencing.

Persons should avoid large gatherings, practice physical distancing and hand hygiene, wear masks in public places, and stay home when ill to protect their family, friends, and the public. Asymptomatic and presymptomatic transmission of SARS-CoV-2 is well documented (*6*,*7*); therefore, persons who have had close contact with confirmed COVID-19 cases should consider being tested.^§§§^ Close contacts should also self-quarantine for 14 days, irrespective of COVID-19-related symptoms.^¶¶¶^ Persons can work with local health officials to increase COVID-19 awareness and determine the best policies for organizing social events to prevent outbreaks in their communities.****

**Summary**

**What is already known about this topic?**

Large gatherings pose a high risk for SARS-CoV-2 transmission.

**What is added by this report?**

A wedding reception with 55 persons in a rural Maine town led to COVID-19 outbreaks in the local community, as well as at a long-term care facility and a correctional facility in other counties. Overall, 177 COVID-19 cases were linked to the event, including seven hospitalizations and seven deaths (four in hospitalized persons). Investigation revealed noncompliance with CDC’s recommended mitigation measures.

**What are the implications for public health practice?**

To mitigate transmission, persons should avoid large gatherings, practice physical distancing, wear masks, stay home when ill, and self-quarantine after exposure to a person with confirmed SARS-CoV-2 infection.

## Footnotes

* https://www.cdc.gov/coronavirus/2019-ncov/downloads/pui-form.pdf.

† https://saraalert.org/.

§ https://www.biorxiv.org/content/10.1101/2020.07.22.216275v1.

¶ 45 C.F.R. part 46.102(l)(2), 21 C.F.R. part 56; 42 U.S.C. Sect. 241(d); 5 U.S.C. Sect, 552a; 44 U.S.C. Sect. 3501 et seq.

** https://www.maine.gov/governor/mills/sites/maine.gov.governor.mills/files/inline-files/EO-57.pdf.

†† https://www.maine.gov/decd/covid-19-prevention-checklists.

§§ https://www.medrxiv.org/content/10.1101/2020.08.01.20166595v1.

¶¶ https://www.cdc.gov/coronavirus/2019-ncov/community/correction-detention/guidance-correctional-detention.html.

*** https://www.medrxiv.org/content/10.1101/2020.04.04.20053058v1.

††† https://www.cdc.gov/coronavirus/2019-ncov/hcp/long-term-care.html.

§§§ https://www.cdc.gov/coronavirus/2019-ncov/symptoms-testing/testing.html.

¶¶¶ https://www.cdc.gov/coronavirus/2019-ncov/if-you-are-sick/quarantine.html.

**** https://www.cdc.gov/coronavirus/2019-ncov/need-extra-precautions/other-at-risk-populations/rural-communities.html.
